# Discriminating Cognitive Stability from Incident Impairment with Verbal Fluency Scores Derived from Sentence Transformers

**DOI:** 10.1002/alz70861_108975

**Published:** 2025-12-23

**Authors:** Justin Bushnell, Fredrick Unverzagt, Virginia G Wadley, Richard E Kennedy, David G. Clark

**Affiliations:** ^1^ Indiana University, Indianapolis, IN USA; ^2^ University of Alabama at Birmingham, Birmingham, AL USA

## Abstract

**Background:**

We evaluated the sensitivity of a novel score, derived from automatically transcribed verbal fluency (VF) tasks, in distinguishing individuals who later develop cognitive impairment from those who do not.

**Method:**

We used longitudinal Six‐Item Screener (SIS) evaluations to identify case and control subjects from the Reasons for Geographic and Racial Differences in Stroke (REGARDS) dataset. Cases of Incident Cognitive Impairment (ICI) were defined as individuals with at least one normal SIS score (> 4) followed by an unbroken series of abnormal scores (<= 4). Controls were defined as those who never failed the SIS. Because we wished to assess the potential prognostic value of a novel VF score, we examined VF word lists along with the number of days before conversion (for ICI) and days to censoring (for controls). Subjects who had a stroke were excluded. We automatically transcribed animal and letter VF tasks and applied a coarse automatic correction to the transcriptions by selecting only valid words contained in a corpus of manually transcribed VF tasks. For each transcription, we obtained a word list embedding using a sentence transformers library for Python. We fit a mixed‐effects Cox proportional hazards model with ICI and number of days to convert (or censor) as the outcome, using a centroid‐based impairment score derived from the embeddings, raw score, and demographic covariates as independent variables. For animal fluency, we had 2,076 case observations and 36,299 control observations. For letter fluency, we had 1,399 case observations and 24,736 control observations.

**Result:**

For both animal and letter fluency analyses, our novel vector‐based scores were significant predictors (animal: β=5.45, *p* <0.05; letter: β=5.50, *p* <0.01) for future ICI after controlling for demographics and VF raw score. Both VF raw scores were significant as well (animal: β=‐0.03, *p* <0.001; letter: β=‐0.03, *p* <0.05).

**Conclusion:**

Sentence transformers offer a straightforward and unbiased method for converting a VF word list into a vector representation containing prognostically relevant information beyond that afforded by raw scores. Future work will assess the predictive value of this additional information.

